# A Simple Near‐Infrared Fluorescent Probe for the Detection of Peroxynitrite

**DOI:** 10.1002/open.201900301

**Published:** 2019-12-09

**Authors:** Luling Wu, Xue Tian, Hai‐Hao Han, Jie Wang, Robin R. Groleau, Paramabhorn Tosuwan, Boontana Wannalerse, Adam C. Sedgwick, Steven D. Bull, Xiao‐Peng He, Tony D. James

**Affiliations:** ^1^ Department of Chemistry University of Bath, BA2 7AY, UK; ^2^ Key Laboratory for Advanced Materials and Joint International Research Laboratory of Precision Chemistry and Molecular Engineering, Feringa Nobel Prize Scientist Joint Research Center, School of Chemistry and Molecular Engineering East China University of Science and Technology 130 Meilong Rd. Shanghai 200237 China; ^3^ Paramabhorn Tosuwan and Boontana Wannalerse Department of Chemistry, Facultry of Science Kasetsart University Bangkok 10900 Thailand; ^4^ Department of Chemistry University of Texas at Austin 105 E 24th street A5300 Autin TX 78712–1224 USA

**Keywords:** near-infrared, fluorescence, boronate, peroxynitrite, probe

## Abstract

Herein, we report the evaluation and synthesis of a reaction based fluorescent probe **DCM‐Bpin** for the detection of Peroxynitrite (ONOO−). **DCM‐Bpin** exhibits selective fluorescence off‐on response for ONOO^−^ over other reactive oxygen species, including H_2_O_2_. Moreover, **DCM‐Bpin** is biocompatible and has been used to visualize exogenous ONOO^−^ in HeLa cells.

## Graphical abstract

**Turn on the light**: A 50‐fold “turn‐on” fluorescence response at 667 nm was observed for **DCM‐Bpin** upon the addition of ONOO^−^ (0–27 equiv.) using an excitation wavelength of 560 nm.

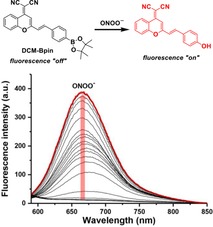

Peroxynitrite (ONOO^−^) is a key intracellular signaling molecule in both physiological and pathological processes, formed *in vivo* by the very fast radical coupling reaction between nitric oxide (NO^.^) and superoxide (O_2_^.−^).^1^ The high reactivity of ONOO^−^ leads to the reaction with almost all types of biomolecules, such as proteins, lipids and DNA, causing oxidative stress and deleterious effects on cellular function.^2^, ^3^ As such, ONOO^−^ has been implicated as a key pathogenic factor for a number of diseases, including inflammatory, ischemia‐reperfusion and neurodegenerative diseases,^4^, ^5^ therefore new and effective technologies for ONOO^−^ detection are of prime importance. Of particular interest is the development of novel small molecule fluorescent probes for the detection of such species, as fluorescence probes are often more selective, less invasive and more convenient than many other methods for the detection of biologically relevant analytes in cells.^6^, ^7^, ^8^ Although a wide array of sensors have been developed with absorption and emission peaks in the visible range (400–650 nm), near‐infrared (NIR) fluorescence sensors (emission in 650–900 nm NIR region) are still rare, despite distinct advantages for *in vitro* and *in vivo* tracing of molecular processes.^9^ Not only does NIR fluorescence avoid interference with the auto‐fluorescence of indigenous molecules, near‐infrared light results in less scattering and deeper tissue penetration, limiting the damage to living cells.^10^, ^11^ As such, the development of NIR fluorescent sensors is of growing interest to the sensing community.^12^, ^13^, ^14^, ^15^, ^16^

Herein we report the evaluation of a D‐π‐A‐based^17^ fluorescent probe **DCM‐Bpin**, in which a 2‐(2‐methyl)‐4*H*‐chromen‐4‐ylidene)malononitrile (**DCM**) serves as the NIR fluorescence acceptor, and the boronate ester Bpin moiety acts as an ONOO^−^ reporter. The fluorescence of the **DCM** system is quenched by Bpin, and so its rapid oxidation to the corresponding phenol (donor) by ROS species can be used to trigger a turn‐on response, resulting in a peroxynitrite‐activated “turn‐on” probe (Scheme 1). **DCM‐Bpin** has previously been reported as part of upconversion nanoparticles (UCNPs) for the detection of hydrogen peroxide, however, the **DCM‐Bpin** probe was not evaluated for the detection of ONOO^−^,^18^ and given that peroxynitrite reacts with boronates much more rapidly,^19^ we reasoned this system would be suitable for the detection of peroxynitrite.

**Scheme 1:**
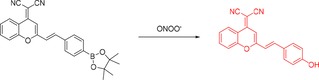
Fluorescence “turn on” mechanism of **DCM‐Bpin** with the addition of ONOO^−^.

The synthesis of **DCM‐Bpin** was successfully achieved in one step by the condensation of 4‐formylphenylboronic acid, pinacol ester and **DCM** (using the previously described procedure).^20^ Heating to reflux in ethanol and piperidine for 4.5 hours, followed by filtration and washing with cold ethanol produced **DCM‐Bpin** as a yellow solid in 62 % yield (Scheme 2).^18^

**Scheme 2:**
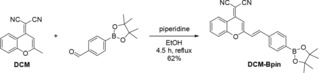
Synthesis of target probe **DCM‐Bpin**.

The UV‐Vis (Figure S1) and fluorescence (Figure 1) behavior of **DCM‐Bpin** was evaluated in pH 7.40 buffer solution (5 % DMSO was required to improve solubility). As shown in Figure S1, the maximum absorption of **DCM‐Bpin** at 434 nm shifted to 530 nm with the addition of ONOO^−^. As expected, **DCM‐Bpin** was initially non‐fluorescent, and upon addition of ONOO^−^ (0‐27 equiv.) a “turn‐on” fluorescence response of up to 50‐fold was observed at 667 nm using an excitation wavelength of 560 nm (Figure 1).

**Figure 1:**
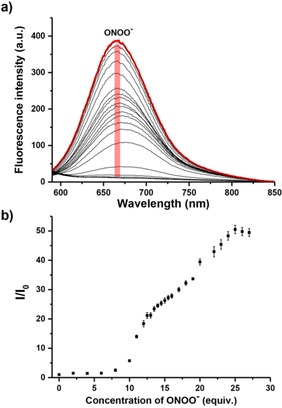
(a) Fluorescence spectra of **DCM‐Bpin** (10 μM) with addition of ONOO^−^ (from 0 to 27 equiv.) in PBS buffer solution (10 mM, containing 5 % DMSO, pH=7.40). The red line shows the highest intensity after addition of ONOO^−^ (25 equiv.); (b) Fluorescence intensity changes (I/I_0_) of probe **DCM‐Bpin** (10 μM) with addition of ONOO^−^ (from 0 to 27 equiv.) in PBS buffer solution (10 mM containing 5 % DMSO, pH=7.40) after 5 min. λ_ex_=560 nm/λ_em_=667 nm. Slit widths: ex=10 nm, em=20 nm.

Subsequently, we compared the response of **DCM‐Bpin** to ONOO^−^ over other reactive oxygen species (ROS), including ClO^−^, ^•^OH, O_2_^.−^, ^1^O_2_, ROO^•^, H_2_O_2_ in PBS buffer (10 mM, pH 7.40). As show in Figures 2 and S2, only the addition of ONOO^−^ to the probe lead to any distinct optical spectral changes, with no “turn‐on” response observed for any of the other ROS species. This indicated that **DCM‐Bpin** has highly selective optical detection for ONOO^−^.

**Figure 2:**
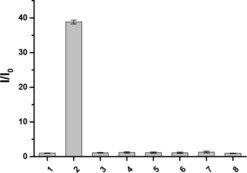
Selectivity bar chart of probe **DCM‐Bpin** (10 μM) with addition of various ROS in PBS buffer solution.1. Blank, 2. ONOO^−^ (200 μM), 3. ^.^OH (500 μM), 4. O_2_^.−^ (500 μM), 5. ^1^O_2_ (500 μM), 6. H_2_O_2_ (500 μM), 7. ROO^.^ (500 μM), 8. ClO^−^ (500 μM). ONOO^−^ were measured after 5 min, other ROS were measured after 1 h. Error bar represents s.d. λ_ex_=560 nm/λ_em_=667 nm. Slit widths: ex=10 nm, em=20 nm.

Having determined the selectivity of **DCM‐Bpin**, we evaluated its ability to image exogenous ONOO^−^ at the cellular level using live HeLa cells. As shown in Figure 3, **DCM‐Bpin** exhibited a good “turn on” response with the addition of peroxynitrite donor SIN‐1.^21^ No fluorescent response was observed in the presence of up to 25 equivalents of H_2_O_2_, confirming the high selectivity of the **DCM‐Bpin** system to ONOO^−^ over other ROS species. Moreover, MTS cell proliferation assays indicated that **DCM‐BPin** was not toxic towards HeLa cells (Figure S5).

**Figure 3:**
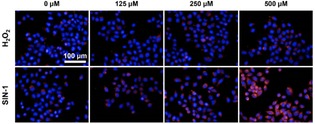
Fluorescence imaging of HeLa cells with **DCM‐Bpin** (20 μM) in the absence or presence of H_2_O_2_ or SIN‐1. Excitation channel=560–580 nm, emission channel=650–760 nm. Cell nuclei were stained with Hoechst 33342.

In conclusion, **DCM‐Bpin**, a near‐infrared fluorescent probe, was shown to be suitable for the detection of ONOO^−^. This probe not only exhibits an excellent “turn‐on” response when exposed to peroxynitrite, it also exhibits high selectivity towards ONOO^−^ over other common reactive oxygen species. **DCM‐Bpin** is biocompatible and displays good sensitivity and selectivity towards ONOO^−^ in HeLa cells.

## Experimental Section

### General Methods

All starting materials and reagents were purchased from Sigma Aldrich, Alfa Aesar, Fluorochem, or Acros Organics, and used as received without any further purification. Unless otherwise stated, all solvents used were reagent grade and were used without distillation. All water was distilled. Thin‐layer chromatography was performed by using commercially available Fluorochem aluminum‐backed plates coated with a layer of silica gel (60 Å) with fluorescent indicator UV254. These plates were visualized by using ultraviolet light with a wavelength of either 254 or 365 nm. Silica gel column chromatography was carried out by using Sigma Aldrich 60 Å silica gel (200–400 mesh).

All NMR spectra were obtained using an Agilent ProPulse 500 with all spectra recorded in chloroform‐*d*. LC−MS analyses were performed using an Agilent QTOF 6545 with Jetstream ESI spray source coupled to an Agilent 1260 Infinity II Quat pump HPLC with 1260 autosampler, column oven compartment and variable wavelength detector (VWD). All pH measurements taken during fluorescence/absorption experiments were recorded on a Hanna Instruments HI 9321 microprocessor pH meter, which was routinely calibrated by using Fisher Chemicals standard buffer solutions (pH 4.0: phthalate; 7.0: phosphate; 10.0: borate). UV‐Vis measurements were performed on an Agilent Cary 60 UV‐Vis Spectrophotometer, utilizing a Hellma silica (quartz) cuvette with a 10 mm path length (provides photometry with two windows). Fluorescence study was performed an Agilent Cary Eclipse Fluorescence Spectrophotometer.

Full synthetic procedures, characterisation data, and fluorescence analysis protocols can be found in the Supporting Information.

## Conflict of interest

The authors declare no conflict of interest.

## Supporting information

As a service to our authors and readers, this journal provides supporting information supplied by the authors. Such materials are peer reviewed and may be re‐organized for online delivery, but are not copy‐edited or typeset. Technical support issues arising from supporting information (other than missing files) should be addressed to the authors.

Supplementary
