# Supplementary material for: A Simple Near‐Infrared Fluorescent Probe for the Detection of Peroxynitrite
Source: ChemistryOpen. 2019 Dec 9;8(12):1407–9. doi: 10.1002/open.201900301 (PMC6900745; doi:10.1002/open.201900301)
Supplement: Supplementary file 1 — Supplementary [file OPEN-8-1407-s001.pdf]

ChemistryOPEN
Open Access

Supporting Information

© Copyright Wiley-VCH Verlag GmbH & Co. KGaA, 69451 Weinheim, 2019

A Simple Near-Infrared Fluorescent Probe for the Detection of Peroxynitrite

Luling Wu[+], Xue Tian[+], Hai-Hao Han, Jie Wang, Robin R. Groleau, Paramabhorn Tosuwan, Boontana Wannalerse, Adam C. Sedgwick, Steven D. Bull, Xiao-Peng He,* and Tony D. James*© 2019 The Authors. Published by Wiley-VCH Verlag GmbH & Co. KGaA. This is an open access article under the terms of the Creative Commons Attribution License, which permits use, distribution and reproduction in any medium, provided the original work is properly cited.An invited contribution to a Special Collection dedicated to Functional Supramolecular Systems

Table of contents

1. UV-Vis and fluorescence analysis S2
2. Generation of various ROS S3
3. Mass spectrometry analysis S4-S5
4. Detailed protocols for cell culture S6
5. Fluorescence imaging in live cells and MTS assay S6
6. Experimental S7
7. NMR spectrum S8-S9
8. References S10
9. Author contributions S10

1. UV-Vis and fluorescence analysis

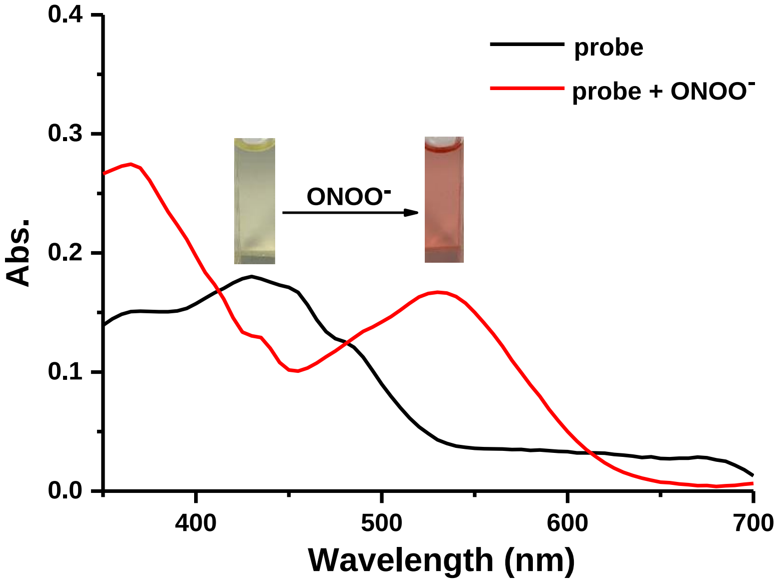

Figure S1. Absorption spectrum of probe **DCM-Bpin** (20 μM) with and without $ONOO^{-}$ (10 equiv.) in PBS buffer solution (containing 5% DMSO, pH = 7.40).

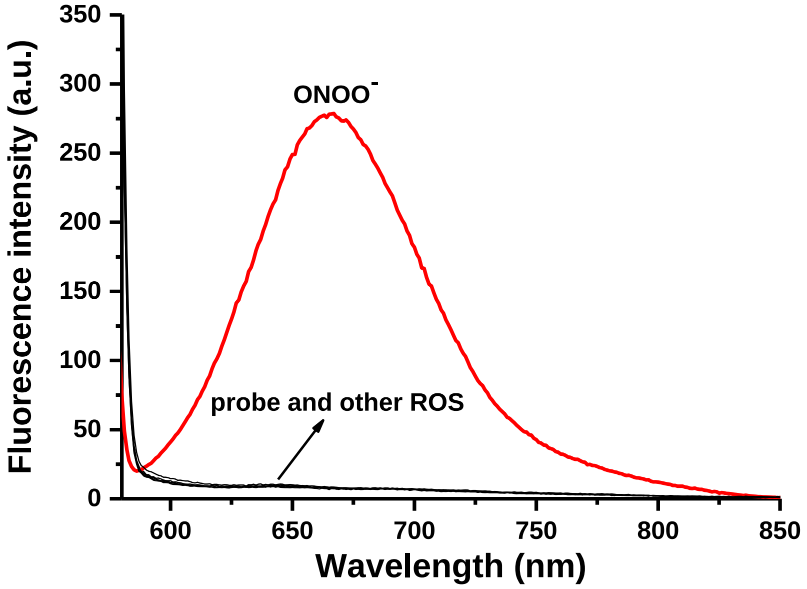

Figure S2. Fluorescence intensity changes of **DCM-Bpin** (10 μM) with addition of $ONOO^{-}$ (200 μM) measured after 5min, and various other ROS (500 μM) measured after 1 h in PBS buffer solution (containing 5% DMSO, pH = 7.40). λ_{ex} = 560 nm. Slit widths: ex = 10 nm, em = 20 nm.

2. Generation of various ROS

ROO$^\bullet$

ROO$^\bullet$ was generated from 2,2'-azobis (2-amidinopropane) dihydrochloride. AAPH (2, 2' azobis (2-amidinopropane) dihydrochloride, 1 M) was added into deionizer water, and then stirred at 37 °C for 30 min.

$O_2^{\bullet-}$

Superoxide was generated from KO_2. KO_2 and 18-crown-6 ether (2.5 eq) were dissolved in DMSO to afford a 0.25 M solution.

$^\bullet OH$

Hydroxyl radical was generated by the Fenton reaction. To prepare $^\bullet OH$ solution, hydrogen peroxide (H_2O_2, 10 eq) was added to $Fe(ClO_4)_2$ in deionised water.

$^1O_2$

$^1O_2$ was generated by reacting H_2O_2 (1 mM) with NaClO (1 mM). The solution of H_2O_2 was added in one portion to the aqueous solution of NaClO and stir for 2 minutes, using the prepared solution immediately.

$ONOO^-$

0.6 M $NaNO_2$, 0.6 M HC1, 0.7 M H_2O_2 was added simultaneously to a 3 M NaOH solution at 0 ºC. The concentration of peroxynitrite was estimated by using extinction co-efficient of 1670 M^{-1} cm^{-1} at 302 nm in a 0.5 M sodium hydroxide aqueous solutions.

ClO^-

The concentration of ClO^- was determined from the absorption at 292 nm (ε = 350 M^{-1} cm^{-1}).

H_2O_2

The concentration of H_2O_2 was determined from the absorption at 240 nm (ε = 43.6 M^{-1} cm^{-1}).

3. Mass spectrometry analysis

Compound Table

Compound Label	RT (min)	Observed mass (m/z)	Neutral observed mass (Da)	Theoretical mass (Da)	Mass error (ppm)	Isotope match score (%)
Cpd 1: C26 H23 B N2 O3	1.01	423.1881	421.1836	421.1838	-0.49	84.76

Mass errors of between -5.00 and 5.00 ppm with isotope match scores above 60% are considered confirmation of molecular formulae

Figure: Extracted ion chromatogram (EIC) of compound.

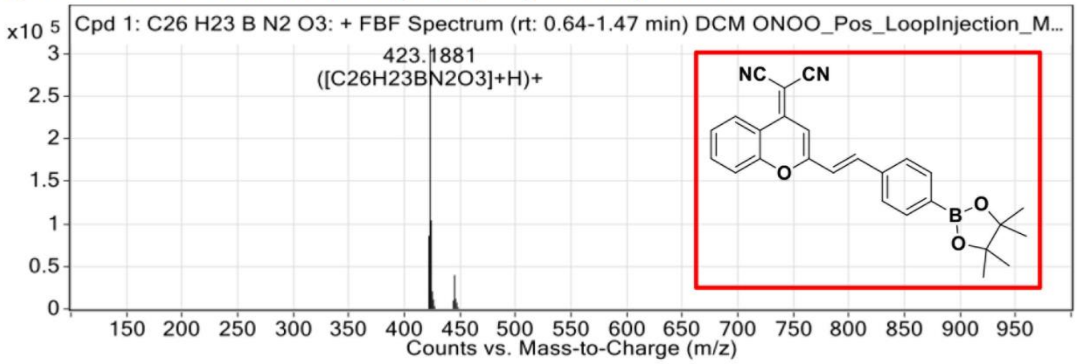

Figure: Full range view of Compound spectra and potential adducts.

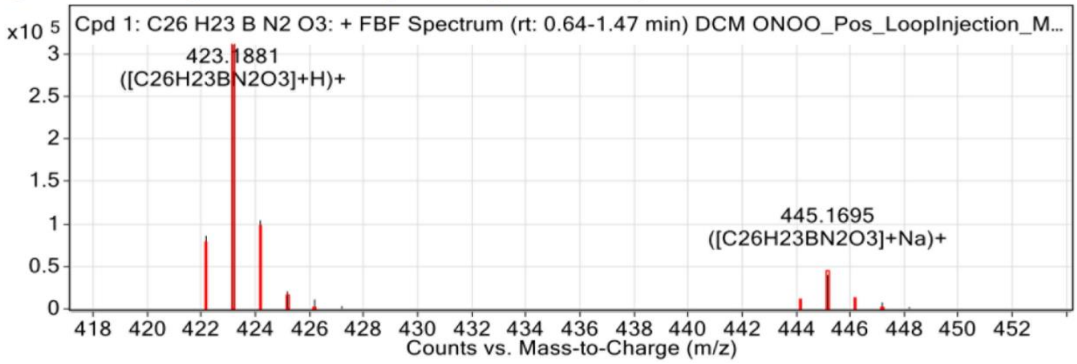

Figure S3. HRMS of probe **DCM-Bpin**.

Compound Table

Compound Label	RT (min)	Observed mass (m/z)	Neutral observed mass (Da)	Theoretical mass (Da)	Mass error (ppm)	Isotope match score (%)
Cpd 1: C20 H11 N2 O2	6.90	312.0927	311.0841	311.0821	6.47	60.89

Mass errors of between -5.00 and 5.00 ppm with isotope match scores above 60% are considered confirmation of molecular formulae

Figure: Extracted ion chromatogram (EIC) of compound.

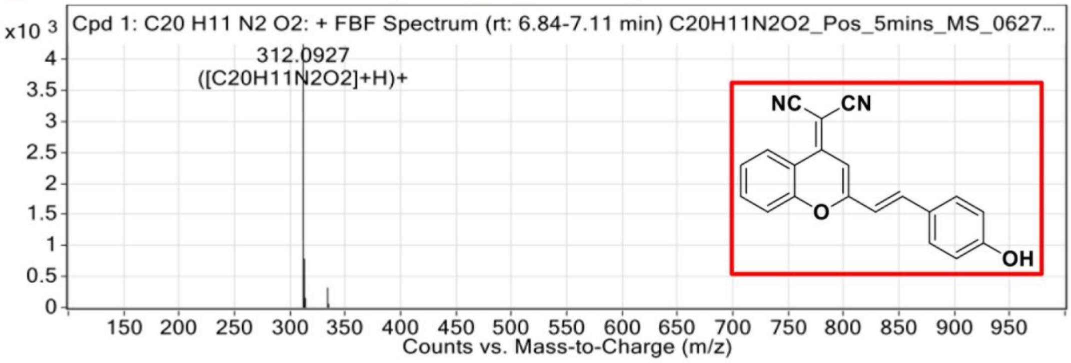

Figure: Full range view of Compound spectra and potential adducts.

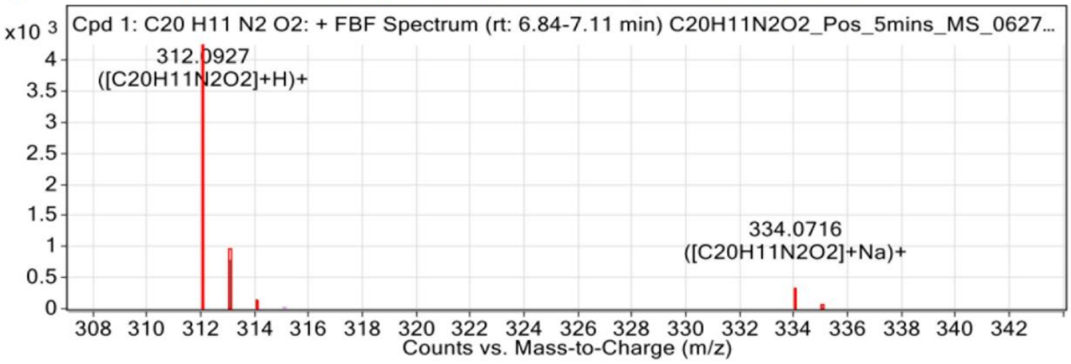

Figure S4. LC-MS of **DCM-Bpin + ONOO⁻**, showing cleavage of Bpin to the related phenol

4. Detailed protocols for cell culture

Cell culture. Hela cells were maintained in a Dulbecco's Modified Eagle's Medium (Invitrogen, Carlsbad, CA, USA) supplemented with 10% fetal bovine serum (Gibco, Gland Island, NY, USA) in a humidified atmosphere of 5% CO_2 and 95% air at 37 °C and split when the cells reached 90% confluency.

5.Fluorescence imaging in live cells and MTS assay

High-content fluorescence imaging. Cells were seeded on a black 96-well microplate with optically clear bottom (Greiner bio-one, Germany) overnight. The cells were incubated with **DCM-Bpin** (20 µM) for 30 min, followed by incubation with SIN-1 (125 µM, 250 µM, 500 µM) or H_2O_2 (125 µM, 250 µM, 500 µM) for 30 min. The cells' nuclei were stained with Hoechst 33342 (5 µg mL^{-1}). Thecells were then washed with PBS (phosphate buffered saline) three times. The fluorescence images (red) were recorded using an Operetta high content imaging system (Perkinelmer, US) at an excitation wavelength of 560–580 nm and an emission wavelength of 650–760 nm and quantified and plotted by Columbus analysis system (Perkinelmer, US). The excitation wavelength of fluorescence images (blue) was 360-400 nm and the emission wavelenth was 410-480 nm.

Cell viability assay. Cells were plated on 96-well plates in growth medium overnight. The cells were treated with **DCM-Bpin** at different concentrations for 24 h. Then, the cell viabilities were determined through a standard MTS cell proliferation assay using 1% DMSO as the control.

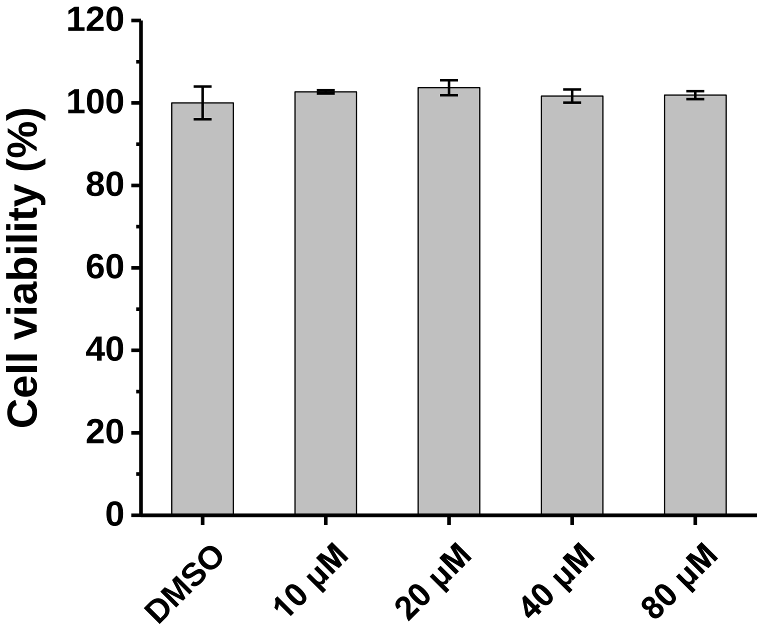

Figure S5. Cell toxicity of **DCM-Bpin** (from 0 to 80 µM) when the incubation time was 24 h. Error bar represents s.d.

6. Experimental

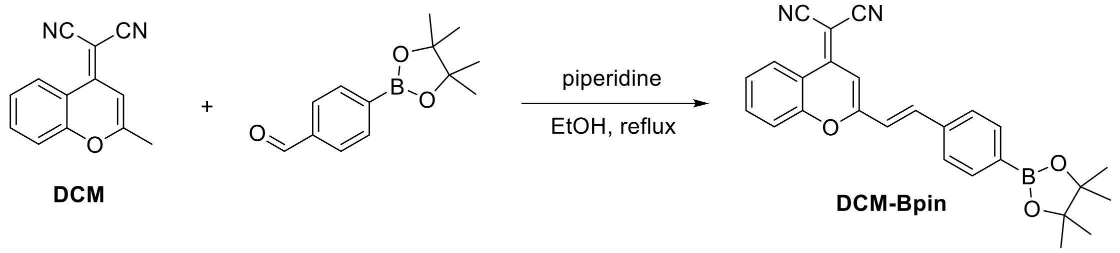

Scheme S1. Synthesis of target probe **DCM-Bpin**.

Synthesis of 2-(2-methyl-4*H*-chromen-4-ylidene)malononitrile (DCM)

DCM was synthesized according to previously reported procedures. Characterisation data were consistent with previous literature reports.[1] M.p. 192 °C; ^{1}H NMR (500 MHz, $CDCl_3$) δ_H 8.91 (dd, J = 8.2, 0.7 Hz, 1H), 7.73-7.70 (m, 1H), 7.47-7.43 (m, 2H), 6.72 (s, 1H), 2.44 (s, 3H); ^{13}C NMR (125 MHz, $CDCl_3$) δ_C 161.82 (s), 153.36 (s), 153.02 (s), 134.71 (s), 126.17 (s), 125.94 (s), 118.77 (s), 117.69 (s), 116.70 (s), 115.58 (s), 105.60 (s), 62.51 (s), 20.62 (s).

Synthesis of (*E*)-2-(2-(4-(4,4,5,5-tetramethyl-1,3,2-dioxaborolan-2-yl)styryl)-4*H*-chromen-4-ylidene)malononitrile (DCM-Bpin)

DCM (0.10g, 0.50 mmol) and 4-(4,4,5,5-tetramethyl-1,3,2-dioxaborolan-2-yl)-benzaldehyde (0.13g, 0.55 mmol) were added in 5 mL of ethanol absolute. Then piperidine (2.00 mmol) was added and the suspension was reflux for 4.5 h. The mixture was cooled and the solid precipitate was filtered and washed with cold ethanol to afford the title compound as a yellow solid (0.13 g, yield 62%). Characterisation data were consistent with previous literature reports.[2] M.p. 221 °C; ^{1}H NMR (500 MHz, $CDCl_3$) δ_H 8.92 (dd, J = 8.4, 1.3 Hz, 1H), 7.87 (d, J = 8.1 Hz, 2H), 7.76-7.73 (m, 1H), 7.64 (d, J = 16.0 Hz, 1H), 7.59 (d, J = 8.0 Hz, 2H), 7.56 (dd, J = 8.4, 1.0 Hz, 1H), 7.48-7.44 (m, 1H), 6.89 (t, J = 8.0 Hz, 2H), 1.36 (s, 12H); ^{13}C NMR (125 MHz, $CDCl_3$) δ_C 157.37 (s), 152.92 (s), 152.48 (s), 138.90 (s), 137.17 (s), 135.60 (s), 134.85 (s), 127.25 (s), 126.16 (s), 126.00 (s) 119.74 (s), 118.76 (s), 117.98 (s), 116.82 (s), 115.70 (s), 107.34 (s), 84.26 (s), 63.39 (s), 25.04 (s).

7. NMR spectra

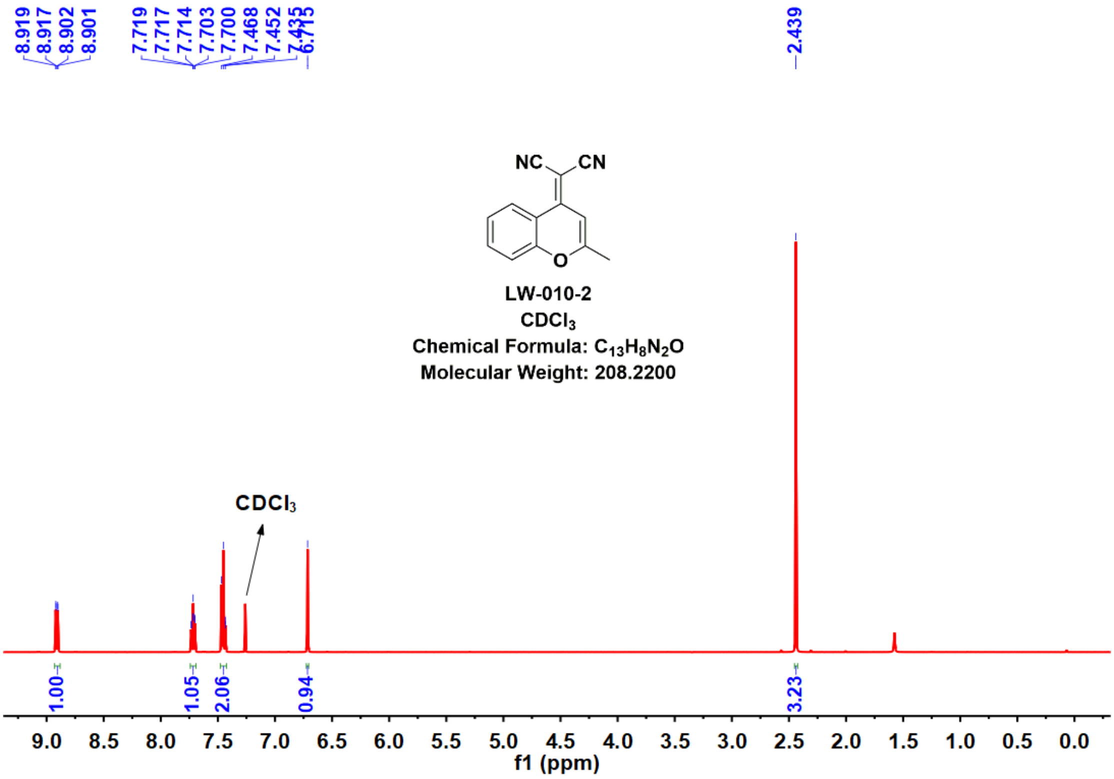

Figure S6. ^{1}H NMR of compound **DCM**.

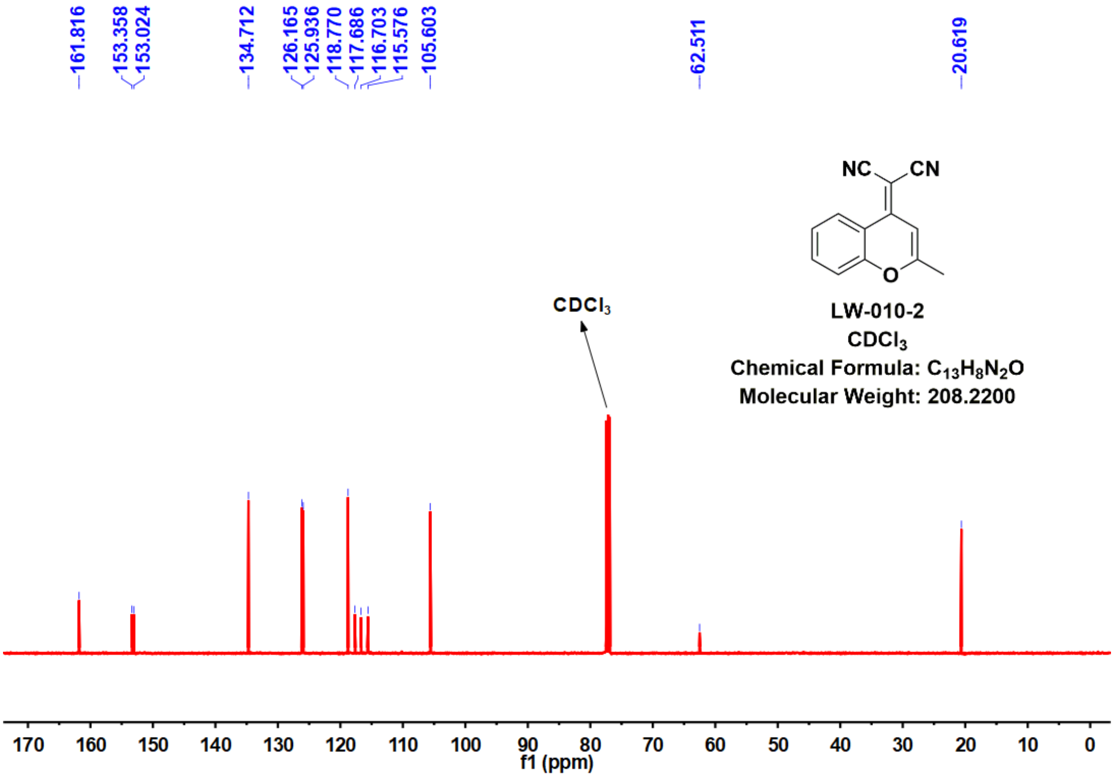

Figure S7. ^{13}C NMR of compound **DCM**.

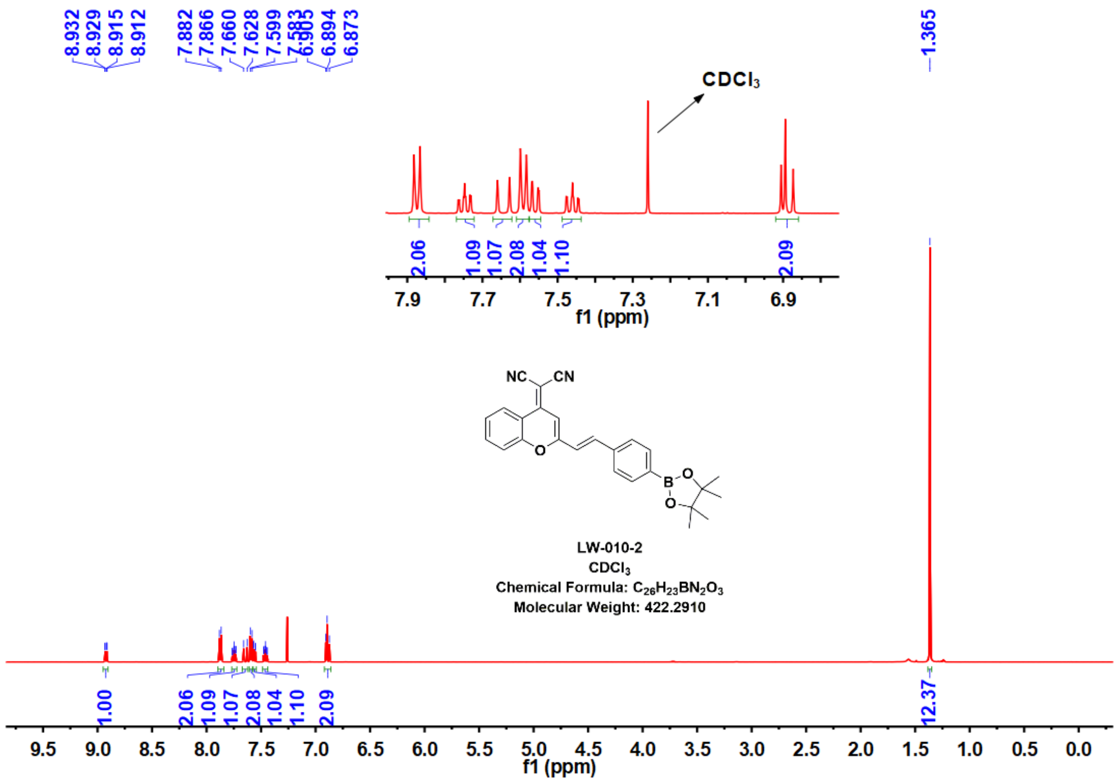

Figure S8. ^{1}H NMR of probe **DCM-Bpin**.

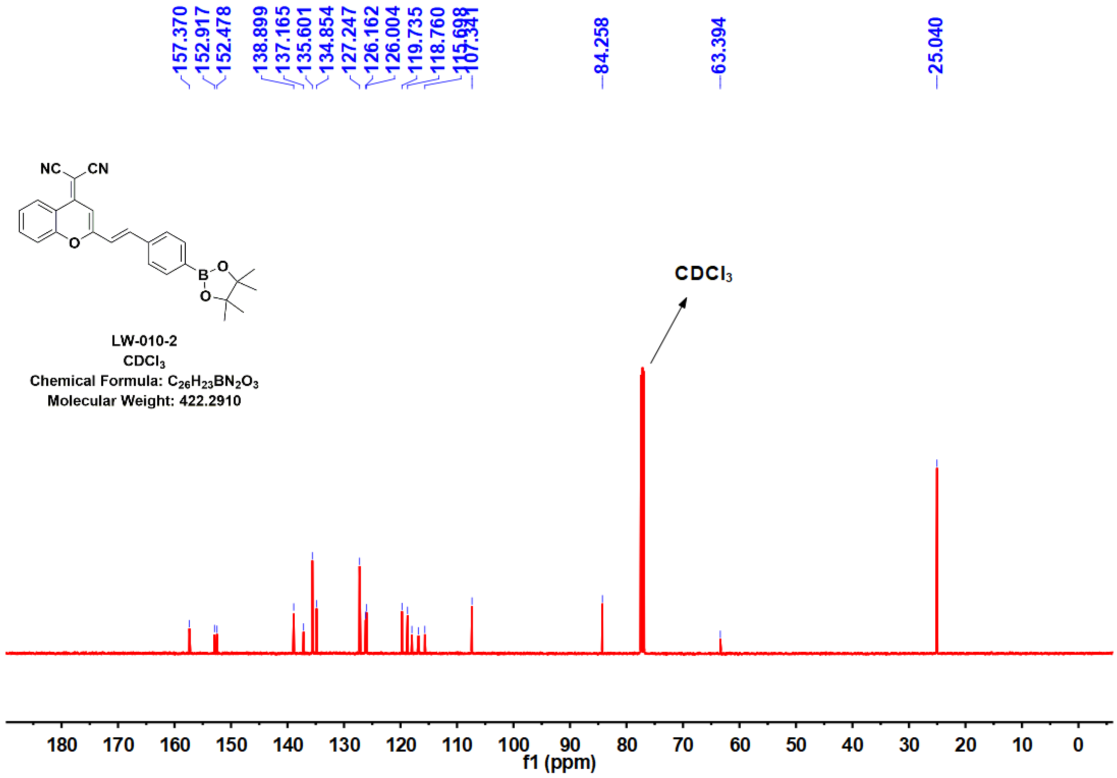

Figure S9. ^{13}C NMR of probe **DCM-Bpin**.

8. References

[1] W. Sun, J. Fan, C. Hu, J. Cao, H. Zhang, X. Xiong, J. Wang, S. Cui, S. Sun, X. Peng, *Chem. Commun.*, **2013**, *49*, 3890.

[2] H. Wang, Y. Li, M. Yang, P. Wang, Y. Gu, *ACS Appl. Mater. Interfaces*, **2019**, *11*, 7441.

9. Author contributions

Luling Wu – wrote the manuscript and synthesized the probe

Xue Tian – wrote the manuscript with Luling Wu and carried out the fluorescence experiments

Hai-Hao Han – carried out the cellular experiments

Jie Wang – helped with the cellular experiments under supervision of Hai-Hao Han

Robin R. Groleau – provided advice and reviewed and edited the manuscript

Paramabhorn Tosuwan – aided Xue Tian in fluorescence experiments

Boontana Wannalerse – supervisor of Paramabhorn Tosuwan

Adam C. Sedgwick – provided advice and reviewed and edited the manuscript

Steven D. Bull – supervisor of Luling Wu, Xue Tian and Robin R. Groleau

Xiao-Peng He – supervisor of Hai-Hao Han and Jie Wang

Tony D. James – lead supervisor
